# Supplementation of quinoa regulates glycolipid metabolism and endoplasmic reticulum stress in the high-fat diet-induced female obese mice

**DOI:** 10.1186/s12986-021-00622-8

**Published:** 2021-10-26

**Authors:** Tian An, Jia-Xian Liu, Xiu-yan Yang, Bo-han Lv, Yan-xiang Wu, Guang-jian Jiang

**Affiliations:** 1 School of Traditional Chinese Medicine, Beijing University of Chinese Medicine, Beijing, China; 2 Zhongli Science and Technology Limited Company, Beijing, 100022 China

**Keywords:** Quinoa, Endoplasmic reticulum stress, Glycolipid metabolism, High-fat diet-induced obesity, Oxidative stress

## Abstract

**Objective:**

To explore the effects of the quinoa diet on glycolipid metabolism and endoplasmic reticulum (ER) stress in an obese mouse model.

**Methods:**

Six-week-old C57BL/6J female mice have received a high-fat diet (HFD) to induce obesity and subsequently were treated with a quinoa diet for 12 weeks. During this period, fasting blood glucose, body fat and insulin resistance were measured regularly. At the end of the experiment, mouse serum and liver tissue were collected. The differences in glucose and lipid metabolism were analyzed, and liver tissue pathological morphology, liver endoplasmic reticulum stress-related mRNA and protein levels, and serum oxidative stress levels were measured.

**Results:**

Quinoa diet could significantly reduce the level of blood glucose, triglyceride, cholesterol, low-density lipoprotein, improve glucose tolerance, as well as improve histological changes of liver tissues in obese mice (*P* < 0.05 or < 0.01). Besides, quinoa could improve oxidative stress indicators such as GSH, and MDA (*P* < 0.05 or < 0.01). Furthermore, quinoa can down-regulate mRNA expression of ER stress markers eIF2α, GRP78, and CHOP in the liver of obese mice (*P* < 0.05 or < 0.01).

**Conclusions:**

Quinoa supplementation can improve glycolipid metabolism, regulate ER stress, and alleviate obesity in HFD-induced mice.

## Introduction

In recent years, obesity, a global public health problem, has continued to spread rapidly [1]. As a chronic metabolic disease, obesity is caused by the interaction of many factors such as diet, environment, and genetics [2], such as intracellular stress and inflammation induced by metabolic disturbance stemmed from overnutrition frequently aggravated by a modern, sedentary lifestyle [3]. Expansion of adipose tissue can cause joint pressure, metabolic abnormalities, organ dysfunction, and increased mortality [4]. The chances of obesity reversibility are closely linked to improving the diagnosis and to timely nutritional interventions [5]. Dietary interventions can help treat obesity and overweight.

Chronic activation of endoplasmic reticulum (ER) stress has been shown to play a significant role in the development of insulin resistance in obesity [6]. ER stress is activated in various tissues under conditions related to obesity, and hepatic ER stress contributes to the development of steatosis and insulin resistance [7]. Furthermore, abnormal lipid accumulation in obesity can also induce ER stress and cellular apoptosis in several tissue types [8]. ER stress is regarded as a central feature of insulin resistance at the molecular, cellular, and organismal levels [9]. Multiple perturbations including inflammation, high glucose, oxidative stress, and high cholesterol can cause ER stress conditions [10]. In addition, Obesity can cause a marked reorganization of mitochondrial-related ER membranes in the liver, leading to mitochondrial calcium overload, impaired mitochondrial oxidative capacity, and increased oxidative stress [11]. Therefore, maintaining ER homeostasis is essential for normal cellular function and survival.

Quinoa (*Chenopodium quinoa Willd.*) is a pseudo-cereal originally cultivated in the Andes Mountains of South America [12]. In recent years, the focus of quinoa seeds has rapidly gained due to its high-quality protein with a wide amino acid spectrum [13]. In addition, the quinoa plant is resistant to cold, salt, and drought, which makes it possible to be widely cultivated all over the world [14]. Consequently, considering the appropriate nutritional and functional properties of quinoa protein, it can be considered a good candidate to supply human food products [15]. Quinoa seeds can produce a variety of secondary metabolites with broad spectra of bioactivities, including saponin, phytosterols, phytoecdysteroids, phenols, and bioactive peptides, which makes them an important candidate for dietary interventions to combat obesity, metabolic syndrome, and T2DM [16, 17].A clinical study showed that a daily intake of 50 g quinoa can reduce serum triglyceride and reduce the prevalence of metabolic syndrome in overweight and obese participants [18]. In addition, animal experiments also showed that quinoa could significantly improve the liver lipid accumulation and glucose homeostasis in obese mice, and normalize HOMA-IR and blood lipid in animals with an altered in glucose and lipid metabolism [19, 20]. Furthermore, cell experiments showed that quinoa polysaccharide significantly inhibited 3T3-L1 differentiation by inhibiting fat transcription factors such as PPARγ, C/EBPα, C/EBPβ, C/EBPδ, SREBP1C, and AP2 expression [21]. However, the specific mechanism of the anti-obesity effect of quinoa is still unclear. Therefore, in this study, we aimed at clarifying the effects of the quinoa diet on a high-fat diet (HFD) induced obesity mice, and the regulation of quinoa diet on ERS, to reveal the mechanisms on the anti-obesity effect of quinoa.

## Methods and materials

### Food and animals

Quinoa was purchased from Shanxi Hao Chen Biological Technology Co., Ltd. (Shanxi, China) committed to the production of quinoa. We use high-tech wall-breaking technology to make quinoa seeds into powder, combine them with water, and then heat them in a microwave oven to remove water, and then make them into quinoa food with the same shape as rat pellet feed. Six-week-old C57BL/6J female mice were supplied by Beijing Vital River Laboratory Animal Technology Co., Ltd. (Beijing, China) and housed in the Animal Facility of Beijing University of Chinese Medicine (BUCM). Standard diet and high-fat diet (HFD, 24% Proteins, 24% fats, and 41% carbohydrates) provided by Beijing HFK Bioscience Co., Ltd. (Beijing, China). During the experiment, all mice were allowed free access to water and chow. The experiments were approved by the animal ethics committee of BUCM.

### Experimental design

After one week of adaptation with general meals and diet balance, 10 C57BL/6J mice were randomly selected and served a standard diet as the normal control group. The other thirty mice received HFD feed for 12 weeks. The obesity model was considered to be successfully constructed if body weight was 20% greater in HFD-fed mice than chow diet-fed mice at the end of this period. Thirty obese mice from the HFD-fed group were selected and randomly subdivided into three groups with ten mice in each. These groups were administered metformin (100 mg/kg body weight/d; metformin group), Quinoa (2 g quinoa/day; Quinoa group), or vehicle (an equivalent volume of water; model control group) by oral gavage daily for 12 weeks. During the experiment, all mice were fed the previous diet. Food intake, body weight, and fasting blood glucose concentrations (FBG) were monitored weekly. At the end of the study, blood samples were collected and the serum was stored for further analysis. Liver tissue was taken, weighed and the liver index was calculated [Liver index = (W_liver_ × 10)/ W_body_)]. Then, part of the liver tissue was fixed with 10% formalin for histological examination. The rest of the liver tissue was immediately frozen in liquid nitrogen and stored at -80 ℃ for subsequent analysis.

### Oral glucose tolerance test (OGTT) and serum analysis

Mice were fasted for 8 h the day before being sacrificed. Glucose (Sinopharm Chemical Reagent, Beijing, China) was administered at a dose of 2 g/kg by gavage, and tail vein blood glucose levels were measured using a glucose meter (Johnson & Johnson Medical, Shanghai, China) at 0, 30, 60, 90 and 120 min. Area under curve (AUC) was calculated as follows: AUC (mmol h L^−1^) = 0.5 × (FBG 0 min + FBG 30 min)/2 + 0.5 × (FBG 30 min + FBG 60 min)/2 + l × (FBG 60 min + FBG 120 min)/2. Serum fasting insulin (FINS), High-density lipoprotein cholesterol (HDL-C), low-density lipoprotein cholesterol (LDL-C), total cholesterol (TC), triglycerides (TG), Malondialdehyde (MDA), Glutathione (GSH), Aspartate aminotransferase (AST), Alanine aminotransferase (ALT), Urea nitrogen (UREA), and uric acid (UA) were determined at the end of the study with an automatic biochemistry analyzer.

### Hematoxylin and eosin (HE) staining and Immunohistochemistry (IHC)

Livers were fixed in 10% neutral buffered formalin (Beijing Yi-li Fine Chemical, Beijing, China), embedded in paraffin, sectioned at 4 μm by rotary microtome, stained with hematoxylin–eosin. After HE staining, hepatic histopathological changes were evaluated using a conventional light microscope (Olympus; Tokyo, Japan). Immunohistochemistry was conducted according to the manufacturer's protocol (Zhonghua Jinqiao Biotechnology Co., Ltd; Beijing, China)). Briefly, paraffin-embedded liver sections were placed in the oven at 37 °C for 12 h, then degreased with xylene and hydrated with gradient ethanol. Sequentially, slides were incubated with the antigen retrieval solution, 3% H2O2 for 10 min, and incubated with a primary antibody eIF2α (1:100), GRP78 (1:100) overnight at 4◦C. After that, the slides for IHC staining were incubated with secondary antibodies for 30 min followed by DAB and hematoxylin staining. Finally, the slides were examined and photographed by laboratory microscopy. The intensity of the positive immunohistochemical reaction was analyzed using Image-Pro Plus 6.0 software and expressed as an IOD value.

### Real-time PCR (RT-PCR)

Total RNA was extracted from liver tissue with TRizol reagent (Invitrogen, CA, USA) according to the manufacturer's protocol. Thereafter, total RNA was reverse-transcribed to cDNA using a cDNA synthesis kit (Thermo Scientific, MA, USA). RT-PCR was performed in 20-μL total volume using Applied Biosystems 7500. All samples were pre-incubated at 95 °C for 10 min and then subjected to 42 amplification cycles (95 °C for 15 s, 60 °C for 60 s). The relative mRNA expression was performed by a comparative method (2^−ΔΔCt^) using β-actin as an internal control. The primers used for the PCR were list in Table 1.

**Table 1:** mRNA primers for quantitative PCR analysis

Primer name	Sequence
eIF2α	Forward 5′-TGGGACGCCTAACCTACAAC-3′
	Reverse 5′-TCATCTGACCAGGAAGGACA-3′
GRP78	Forward 5′-GTGTGTGAGACCAGAACCGT-3′
	Reverse 5′-ACAGTGAACTTCATCATGCCG-3′
CHOP	Forward 5′-TATCTCATCCCCAGGAAACG-3′
	Reverse 5′-ATGTGCGTGTGACCTCTGTT-3′

### Western blot analysis

Total proteins were extracted from livers with Whole Cell Lysis Assay (KeyGEN BioTECN, KGP250, China) and the protein concentration was determined by BCA protein assay kit. Subsequently, denatured protein samples were loaded onto the 10% SDS-PAGE gel and transferred to a PVDF membrane of appropriate size. The membrane was incubated with corresponding primary antibodies [GRP78 (1:1000), CHOP (1:500), p-elf2α (1:500)] at 4 °C overnight. The membranes were subsequently incubated with the appropriate secondary antibodies (1:8000) for 1 h at room temperature. The bands were visualized with enhanced chemiluminescence fluid and the images were captured with Azure Bio-imaging systems (CA, USA). Band densities were quantified using Image J software (National Institutes of Health, Bethesda, MD, USA), and normalized with the corresponding β-actin as the internal control.

### Statistical analysis

Data were analyzed using Prism 6.0 and all results were expressed as mean ± standard deviation (SD). A one-way analysis of variance was performed between multiple groups when the data meets the homogeneity of variance and normality. In addition, Dunnett's T3 test is used when the data conforms to a normal distribution but the variance is not uniform. Finally, when the data did fit the normal distribution, nonparametric analysis was used. Differences were considered significant if *P* < 0.05.

## Results

### Supplementation of quinoa decreased the body weight, food intake, and liver index in obese mice

**Fig. 1:**
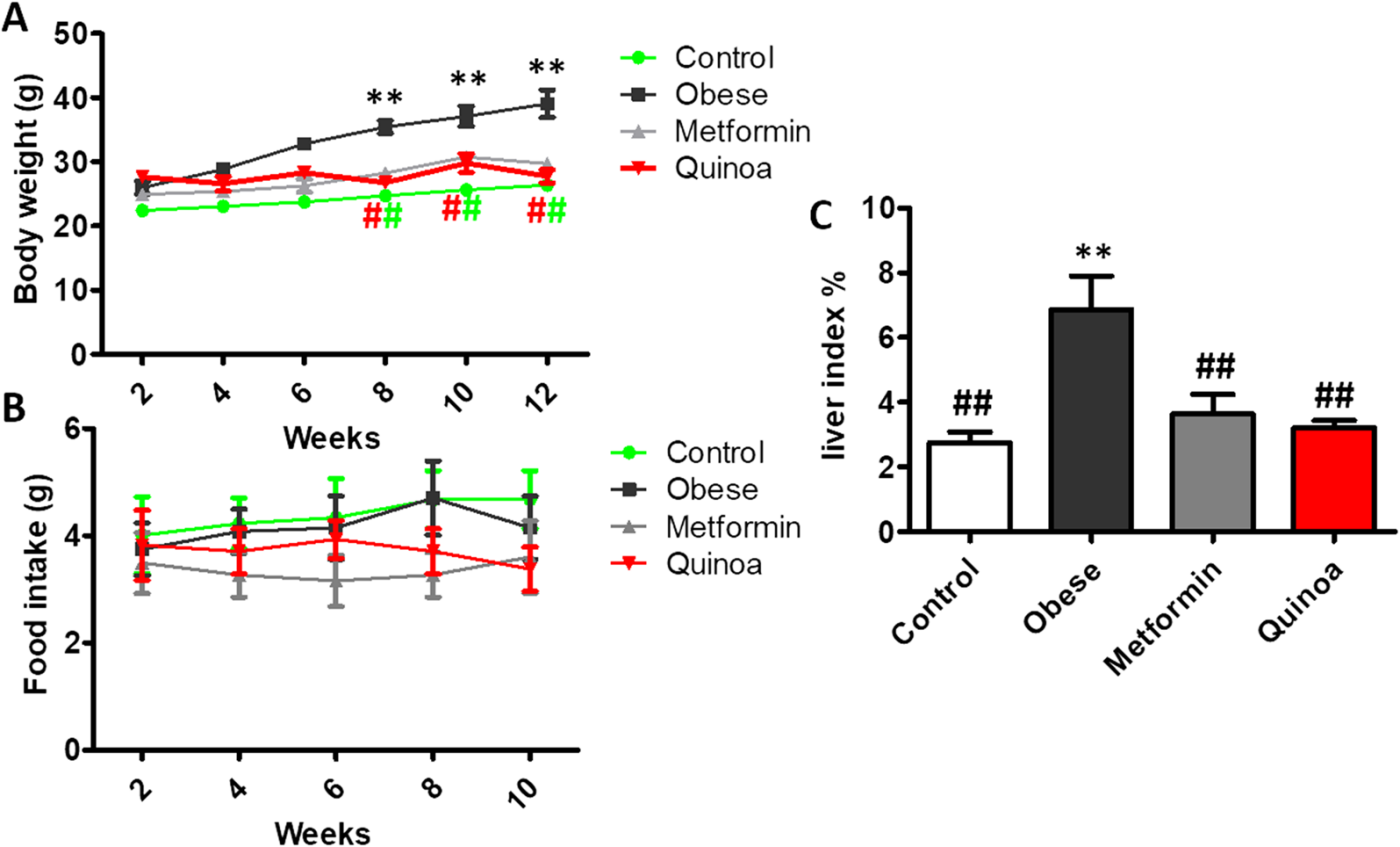
Quinoa decreased the body weight, food intake, and liver index in obese mice. **A** Bodyweight, **B** Food intake, and **C** Liver index. Data are expressed as mean ± SD (n = 6). **P < 0 .01 versus control group; #*P* < 0 .05, ##*P* < 0 .01 versus obese model group. Control: normal mice

To observe the inhibitory effect of Quinoa on obesity, mice were first fed HFD for 12 weeks and then were fed with Quinoa at 2 g/kg body weight for 12 weeks. The body weight of mice with obesity was higher than that of the control group mice (Fig. 1A). After 12 weeks of Quinoa and Met treatment, the body weight and weight gain of mice were significantly decreased (*P* < 0.01, Fig. 1A). Simultaneously, the treatment with Quinoa effectively reduced the liver index and the food intake in obese mice (Fig. 1C). Compared with that in the obesity model group, the food intake in the Quinoa groups decreased from week 6 to week 10 (*P* < 0.01, Fig. 1B). We found that Quinoa significantly reduced the body weight, food intake, and liver index in mice with obesity compared with those in the model group mice.

### Effects of quinoa on serum glucose profile in the obese mice

**Fig. 2:**
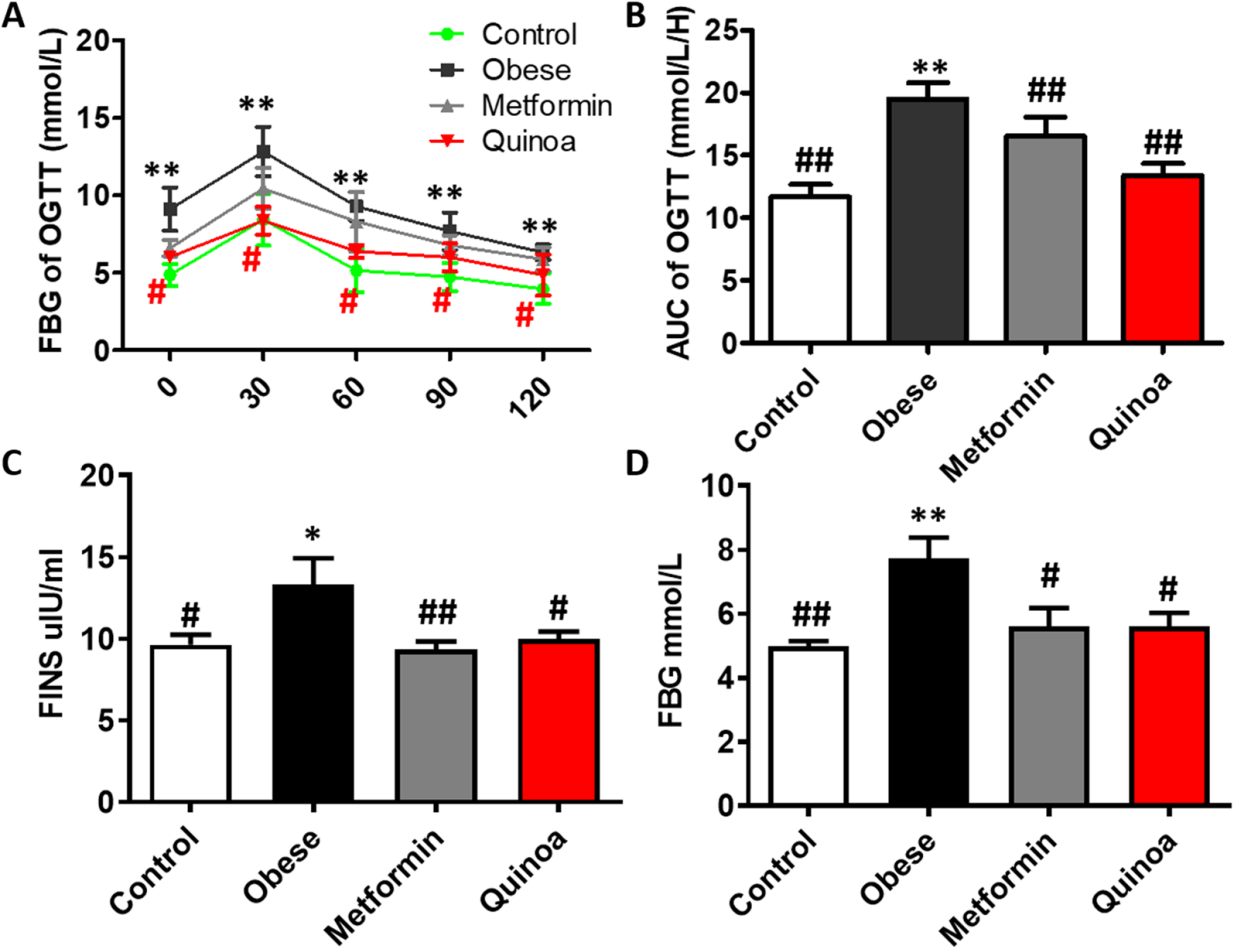
Effect of Quinoa on FBG, OGTT, and FINS in the obese mice. **A** Results of the oral glucose tolerance test (OGTT) by the group at week 12. **B **The area under the OGTT glucose curve by the group at week 12. Quinoa can reduce serum Fasting serum insulin (FINS) levels compared with the obese model group (**C**). **D** Fasting blood glucose (FBG) concentrations. Values are expressed as mean ± SD (n = 6). *P < 0.05, **P < 0.01 versus control group; #*P* < 0.05, ##*P* < 0.01 versus obese group. OGTT: oral glucose tolerance test; AUC: area under the curve

The FBG levels were significantly higher in the model control group than the normal control group during the experiment. Quinoa and metformin treatments significantly reduced the FBG of obese mice relative to the model control group (*P* < 0.05) (Fig. 2D). Figure 2A, B shows the results of the OGTT performed at week 12. Quinoa and metformin treatments significantly decreased the blood glucose levels before and 30, 60, 90, and 120 min after glucose administration relative to the model control group (*P* < 0.05), achieving similar levels to those of the normal control group. The AUC of the Quinoa and metformin groups was significantly smaller than that of the model control group at weeks 6 and 12 (*P* < 0.05). The results indicate that Quinoa lowered the FBG and improved the insulin sensitivity of the obese mice. As shown in Fig. 2C, the serum FINS level of the obese mice was significantly higher than that of the normal mice (*P* < 0.01). After 12 weeks of quinoa treatment, the serum FINS and FBG levels were determined (*P* < 0.01, Fig. 2D). Administration of quinoa for 12 weeks enhanced insulin sensitivity.

### Effects of quinoa on the serum lipid profile and fat mass in the obese mice

**Fig. 3:**
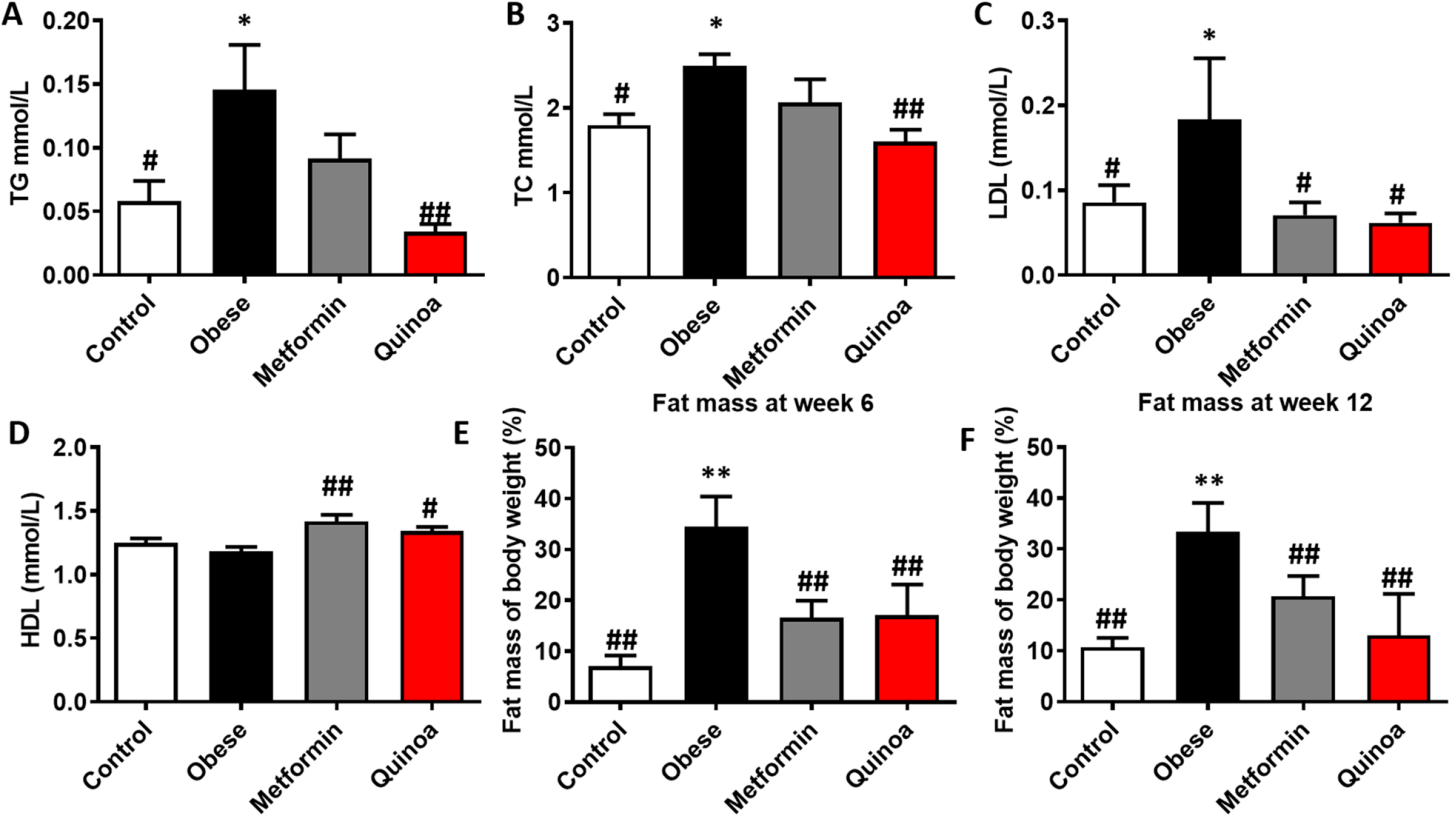
Effects of Quinoa on the serum lipid profile and fat mass in the obese mice. Comparison between treatment groups of serum concentrations of **A** triglyceride; **B** total cholesterol; **C** low-density lipoprotein cholesterol (LDL-C); **D** high-density lipoprotein cholesterol (HDL-C); and percentage body fat mass at **E** week 6 and **F** week 12. Values are expressed as mean ± SD (n = 6). *P < 0.05, **P < 0.01 versus control group; #*P* < 0.05, ##*P* < 0.01 versus obese group

Serum TG, TC, and LDL-C concentrations were significantly higher in the obesity model group than in the control group. Quinoa and Metformin treatment decreased the serum TG, TC, and LDL-C concentrations (Fig. 3A–C) relative to the model control group. Serum HDL-C concentrations in the quinoa and metformin groups were increased relative to the model control group (*P* < 0.01, Fig. 3D).

At weeks 6 and 12, the model control group had approximately twice as much fat as the normal control group (*P* < 0.05). At 6 and 12 weeks, quinoa treatment reduced fat mass relative to the model control group (*P* < 0.05). Compared to the model control group, metformin administration also significantly reduced fat mass (*P* < 0.05) (Fig. 3E, F). The results indicate that quinoa inhibits fat accumulation in HFD-fed obese mice in a time-dependent manner.

### Effect of quinoa on the oxidative stress, liver functions in the obese mice

**Fig. 4:**
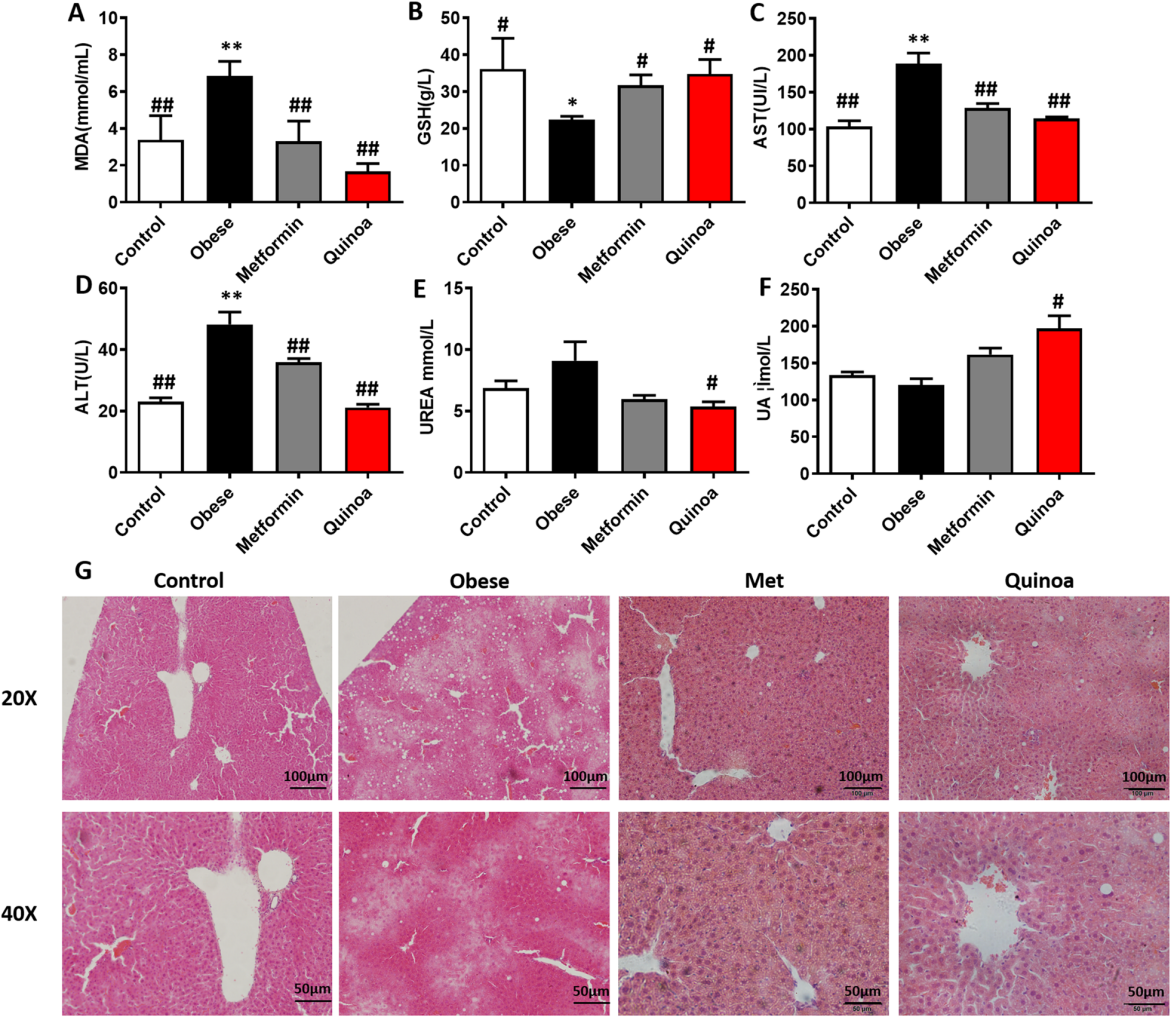
Effect of Quinoa on the Oxidative Stress, Liver functions in the obese mice. **A** to **F** The alteration of Malondialdehyde (MDA), Glutathione (GSH), Aspartate aminotransferase (AST), Alanine aminotransferase (ALT), Urea nitrogen (UREA), and uric acid (UA) in the serum. **G** H&E Staining of hepatic tissue. Representative histology images in each group were exhibited (20, 40 ×). Obese-induced liver injury can be improved by Quinoa intervention. Values are expressed as mean ± SD (n = 6). *P < 0.05, **P < 0.01 versus control group. #*P* < 0.05, ##*P* < 0.01 versus obese group

As shown in Fig. 4A, MDA content in the serum of obese mice induced by HFD was significantly higher than that of normal mice (*P* < 0.05), while GSH activities were lower than those of normal mice (*P* < 0.05, 4B). After 12 weeks of intervention, quinoa effectively reduced serum MDA content (*P* < 0.05) and increased GSH activity (*P* < 0.01). In addition, we observed the effect of quinoa on liver and kidney function in obese mice. As shown in Fig. 4C, D, liver function-related indicators in obese mice were significantly higher than those in normal mice (*P* < 0.05). After 12 weeks of administration, both the quinoa group and metformin group can significantly reduce the serum ALT and AST content (*P* < 0.05), suggesting that quinoa supplement can reduce liver cell damage to a certain extent. A similar phenomenon was also found in the kidney (Fig. 4E, F). After 12 weeks of intervention, quinoa could significantly reduce the levels of urea nitrogen and creatinine in the serum and showed good kidney protection while anti-obesity.

The results of liver HE staining are shown in Fig. 4G. In the normal mice, the structure of the hepatic lobule was clear, and the hepatic cord was arranged in the center of the central vein. Liver cells were regular and polygonal. Compared with the liver of normal mice, the liver architecture of drug-untreated mice with obese showed an increased number of lipid droplets associated with hepatocyte hypertrophy, lymphocyte infiltration, and microvascular steatosis. After 12 weeks of treatment with Quinoa, the histopathological manifestations of the liver tissue were improved.

### Phosphorylated eIF2α and GRP78 expression in the liver IHC

**Fig. 5:**
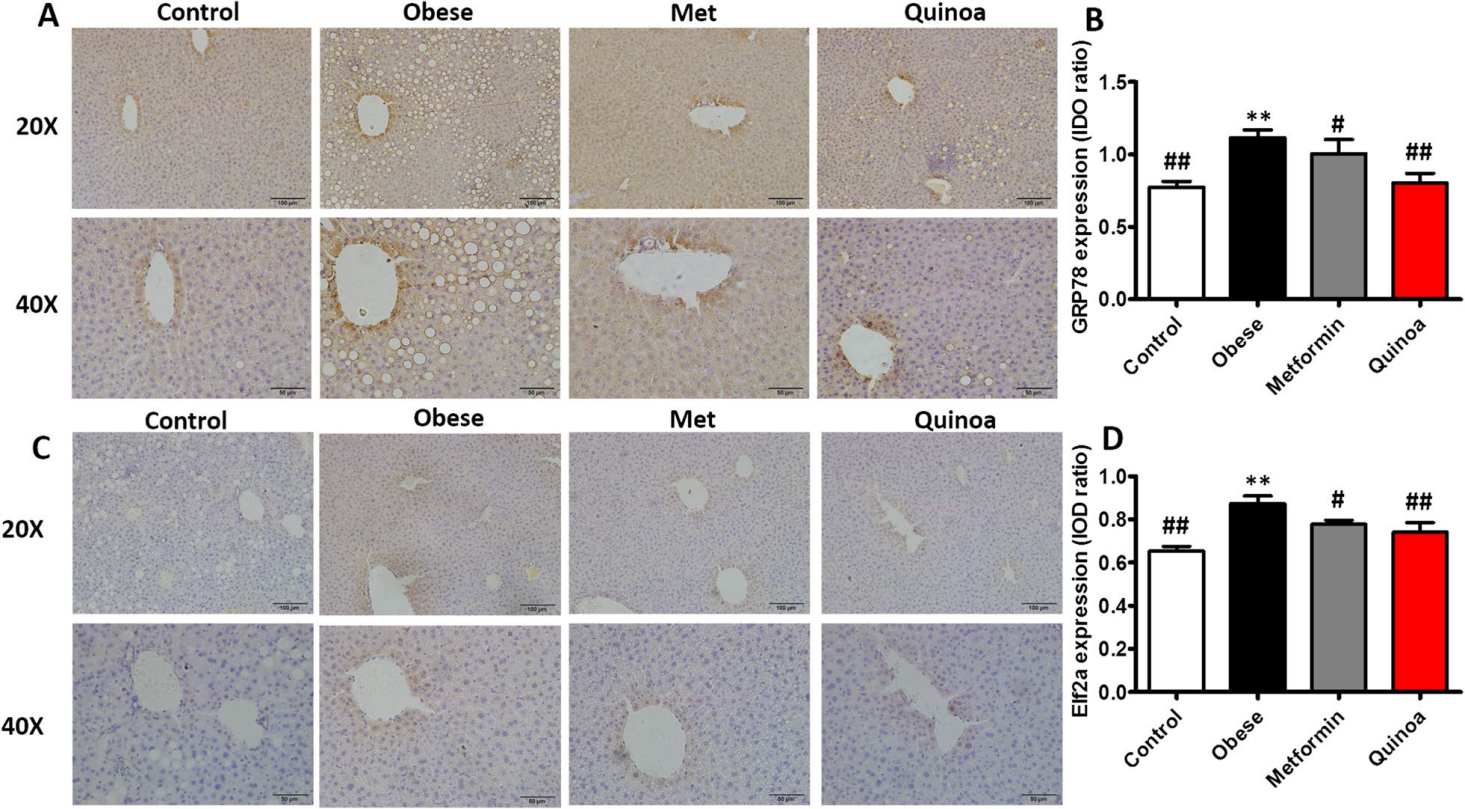
Quinoa can decrease the expression of GRP78 and p-eIF2a compared with the obese model group. Representative immunohistochemical and the analyses of GRP78 (**A**, **B**) and p-eIF2a (**C**, **D**) in the control and obese mice group. Values are expressed as mean ± SD. **P < 0.01 versus control group. #*P* < 0.05, ##*P* < 0.01 versus obese group

The expression of GRP78 (Fig. 5A, B) and p-eIF2α (Fig. 5C, D) was observed in the four groups. Compared with that in the normal ICR mice, the expression of p-eIF2α (Fig. 5D) and GRP78 (Fig. 5B) was increased in the liver of mice with obesity. After 12 weeks of quinoa treatment, the expression of eIF2α and GRP78 in the liver IHC was decreased.

### Expression of phosphorylated p-eIF2α, GRP78, and CHOP proteins in obese mice

**Fig. 6:**
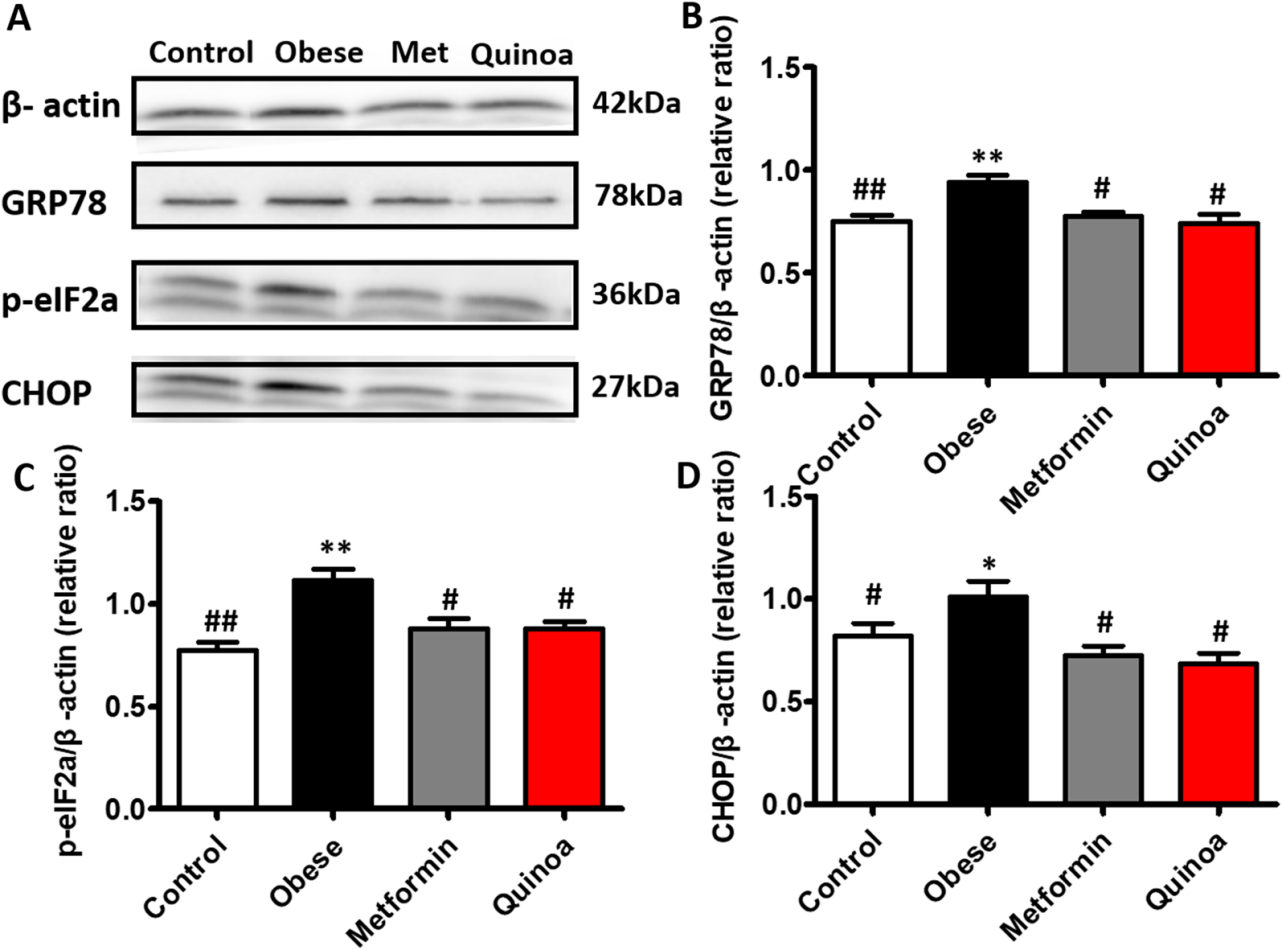
Effect of quinoa on the expressions of p-eIF2α, GRP78, and CHOP proteins in the livers of obese mice. Quinoa can reduce the expression of p-eIF2a, GRP78, and CHOP proteins compared with the obese model group. Values are expressed as mean ± SD (n = 6). *P < 0.05, **P < 0.01 versus control group. #*P* < 0.05, ##*P* < 0.01 versus obese group. p-eIF2α: phosphorylated eukaryotic initiation factor-2 alpha; GRP78: glucose-regulated protein 78; CHOP: C/EBP homology protein

The expression of p-eIF2α, GRP78, and CHOP in the mice with obesity were higher than those in the normal mice (Fig. 6). After treatment with quinoa, the expression of all ER stress markers was reduced compared with that in the drug-untreated mice with T2DM. However, the protein expression of eIF2α exhibited a trend toward reduction, but without statistical significance.

### Effect of quinoa on the mRNA expression of ER stress markers in obese mice

**Fig. 7:**
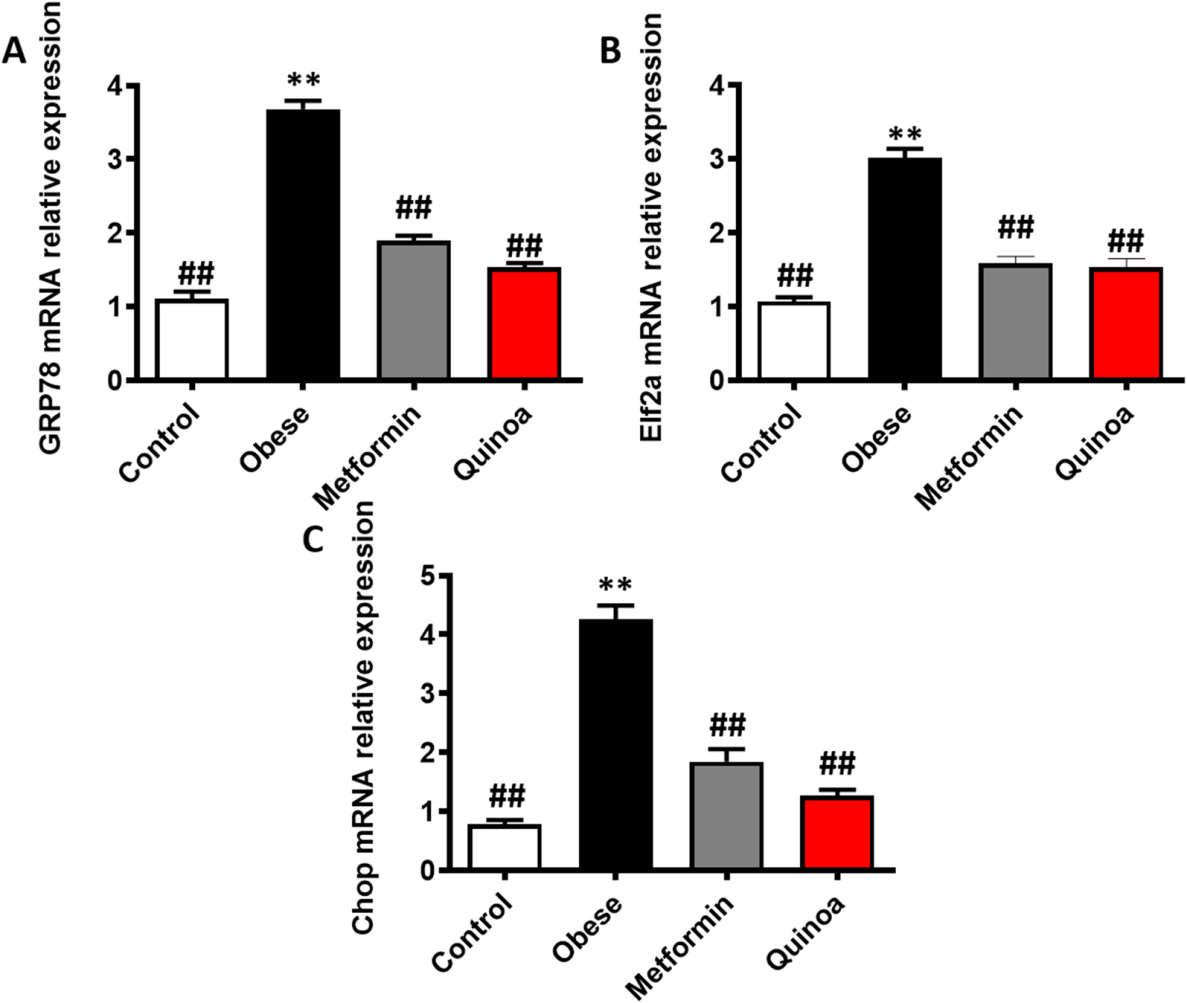
Compared with the obese model group, quinoa can reduce the expression of mRNA associated with ER stress in the liver. Representative western blot images and the analyses of GRP78 (**A**), eIF2α (**B**), and CHOP (**C**). Values are expressed as mean ± SD (n = 6). ##*P* < 0.01 versus obese group. **P < 0.01 versus control group. eIF2α: eukaryotic initiation factor-2 alpha; GRP78: glucose-regulated protein 78; CHOP: C/EBP homology protein

As shown in Fig. 7, the relative mRNA expression of GRP78, eIF2α, and CHOP in the obese model group was significantly higher than that in the control group (*P* < 0.01). Quinoa treatment of mice with obesity reduced the gene expression of eIF2α, GRP78, and Chop compared with that in drug-untreated mice with obesity. Particularly, treatment with a low dose of Quinoa for 12 weeks significantly decreased the relative expressions of eIF2α, GRP78, and CHOP (*P* < 0.01).

## Discussion

As the huge health and economic burden caused by obesity are not seen as urgent enough to generate public demand, the prevalence of adiposity is increasing in every region of the world [22]. "The Lancet" studies have shown that China's obese population has surpassed the United States and ranks first in the world, meanwhile the proportion of female obese patients far exceeds that of male obese patients [23]. If present trends continue, by 2025, global obesity prevalence will surpass 21% in women. Obesity is associated with many negative influences on a female's health, such as infertility [24], polycystic ovary syndrome [25], breast cancer [26], and other adverse maternal and fetal effects prenatally. Therefore, finding strategies to address women's obesity is particularly significant and challenging. C57BL/6J mice can serve as a polygenic developmental model for diet-induced obesity [27]. Overnutrition can cause insulin resistance, leading to obesity [28]. Therefore, in this study, we used C57BL/6J female mice to induce an obesity model by feeding a high-fat diet for 12 weeks. At the same time, quinoa seeds were given as a supplementary diet, and its anti-obesity mechanism was explored by measuring the body weight, body fat, glucose, and lipid metabolism expression profile, and oxidative stress and endoplasmic reticulum stress-related factors in obese mice.

In this study, we found that Quinoa supplementation could significantly improve symptoms of HFD-induced obesity and the correlated indices, as well as repair the impaired glucose tolerance in obese mice, showing an anti-obesity effect. These results are consistent with a previous report [18]. Abnormal lipid metabolism and weight gain are unavoidable in HFD-induced obesity mice [29]. Our results suggest that Quinoa intervention can down-regulate TG, TC, and LDL levels while up-regulating HDL levels, as previously described [19] Quinoa showed a significant regulatory effect on blood glucose and blood lipid levels, and the Quinoa intervention group showed regulation of glycolipid metabolism. In addition, our study also found that Quinoa intervention has a high potential to increase glucose tolerance. These results indicate that Quinoa intervention has beneficial hypoglycemic and hypolipidemic effects in the HFD-induced mice.

Oxidative stress is the main mechanism throughout the development of obesity and its complications [30]. Under normal physiological conditions, ROS produced in the body can be dynamically balanced by the anti-oxidative stress defense system including SOD, CAT, and GSH [31]. Studies have found that obesity can affect the body's oxidative stress levels in a variety of ways, including energy metabolism, and mitochondrial electron transport chain, leading to increased ROS and causing ER stress [32]. In the present study, compared with normal mice, the expression of GSH and SOD in the obese mice induced by HFD was significantly decreased while the content of MDA was significantly increased. Quinoa interventions significantly increased the activity of SOD and GSH antioxidants in obese mice and decreased the expression of MDA. This study suggests that Quinoa has good anti-oxidant properties and Quinoa improves liver dysfunction may be related to anti-oxidation.

Oxidative stress often induces ER stress, as there is tight crosstalk between the two; for example, the ROS produced by disulfide bond formation causes both oxidative and ER stress [33]. Numerous lines of evidence support the involvement of ER stress in the dysregulation of lipid metabolism in obesity [34, 35]. The classical ERS pathway consists of three distinct branches: activation of transcription factor 6 (ATF6), protein kinase R-like endoplasmic reticulum kinase (PERK), and inositol-requiring enzyme 1 (IRE1) [36]. The massive activation of GRP78 helps protein fold to form the correct morphology and regulate the homeostasis of the endoplasmic reticulum [37]. Increased expression of GRP78 and GHOP protein are two important markers of endoplasmic reticulum stress [38]. Activation of PERK can phosphorylate eIF2α, and phosphorylation of eIF2α can reduce the transcription and translation of cells, thereby reducing protein production [39]. Therefore, activation of the PERK/p-eIF2α pathway can reduce protein synthesis in the endoplasmic reticulum and regulate endoplasmic reticulum homeostasis to reduce stress in the ER. In the current study, the expression of GRP78, eIF2α, and GHOP in the liver tissue of the obesity model group was significantly higher than that of the normal group. After Quinoa intervention, the protein expression of GRP78, eIF2α, and GHOP in the liver tissue of obesity mice was significantly decreased, which indicating that Quinoa can down-regulate the expression of GRP78, eIF2α, and GHOP in the liver tissue of obese mice, inhibiting the ERS, and exerting the anti-obesity effect.

## Conclusions

Collectively, our results demonstrate high-fat consumption alters ER homeostasis in the liver. Furthermore, our study supports Quinoa treatment to reduce body weight and body fat mass and improve serum glucose and lipid distribution in a mouse model of HFD-induced obesity. The underlying mechanisms involve the regulation of ER stress through eIF2α, GRP78, and CHOP by Quinoa in liver tissues. We suggest that quinoa, therefore, has the potential to be a therapeutic agent for obesity and its associated metabolic abnormalities, including hyperlipidemia, hyperglycemia, and insulin resistance.

## Data Availability

The datasets used and analyzed during the current study are available from the corresponding author on reasonable request.

## Abbreviations

ER: Endoplasmic reticulum
HFD: High fat diet
FBG: Fasting blood glucose
OGTTs: Oral glucose tolerance tests
FINS: Fasting insulin
ISI: Insulin sensitivity index
GRP78: Glucose regulated protein 78
eIF2α: Eukaryotic initiation factor-2 alpha
ATF4: Activating transcription factor 4
CHOP: C/EBP homology protein
NASH: Nonalcoholic steatosis
NTDs: Neural tube defects
AUC: Area under curve
HE: Hematoxylin eosin
IHC: Immunohistochemistry
RT-PCR: Real-time PCR
ANOVA: Analysis of variance
PERK: PKR-like ER kinase
IRE1α: Inositol-requiring kinase 1α
ATF6: Activating transcription factor 6
